# Brain Activation in Response to Low-Calorie Food Pictures: An Explorative Analysis of a Randomized Trial With Dapagliflozin and Exenatide

**DOI:** 10.3389/fendo.2022.863592

**Published:** 2022-05-04

**Authors:** Charlotte C. van Ruiten, Dick J. Veltman, Max Nieuwdorp, Richard G. IJzerman

**Affiliations:** ^1^ Diabetes Center, Department of Internal Medicine, Amsterdam University Medical Center, Vrije University Medical Center (VUmc), Amsterdam, Netherlands; ^2^ Department of Psychiatry, Amsterdam University Medical Center, Vrije University Medical Center (VUmc), Amsterdam, Netherlands; ^3^ Department of Vascular Medicine, Amsterdam University Medical Center (AMC), Amsterdam, Netherlands

**Keywords:** SGLT2 inhibitor, dapagliflozin, GLP-1 receptor agonist, exenatide, functional neuroimaging, type 2 diabetes, obesity, central regulation of food intake, low-calorie

## Abstract

**Background and Aim:**

Sodium-glucose cotransporter-2 inhibitors (SGLT2i) induce less weight loss than expected. This may be explained by SGLT2i-induced alterations in central reward and satiety circuits, contributing to increased appetite and food intake. This hyperphagia may be specific to high-calorie foods. Glucagon-like peptide-1 receptor agonists (GLP-1RA) are associated with lower preferences for high-calorie foods, and with decreased activation in areas regulating satiety and reward in response to high-calorie food pictures, which may reflect this lower preference for energy-dense foods. To optimize treatment, we need a better understanding of how intake is controlled, and how [(un)healthy] food choices are made. The aim of the study was to investigate the effects of dapagliflozin, exenatide, and their combination on brain activation in response to low-calorie food pictures.

**Methods:**

We performed an exploratory analysis of a larger, 16-week, double-blind, randomized, placebo-controlled trial. Sixty-eight subjects with obesity and type 2 diabetes were randomized to dapagliflozin, exenatide, dapagliflozin plus exenatide, or double placebo. Using functional MRI, the effects of treatments on brain responses to low-calorie food pictures were assessed after 10 days and 16 weeks.

**Results:**

Dapagliflozin versus placebo decreased activity in response to low-calorie food pictures, in the caudate nucleus, insula, and amygdala after 10 days, and in the insula after 16 weeks. Exenatide versus placebo increased activation in the putamen in response to low-calorie food pictures after 10 days, but not after 16 weeks. Dapagliflozin plus exenatide versus placebo had no effect on brain responses, but after 10 days dapagliflozin plus exenatide versus dapagliflozin increased activity in the insula and amygdala in response to low-calorie food pictures.

**Conclusion:**

Dapagliflozin decreased activation in response to low-calorie food pictures, which may reflect a specific decreased preference for low-calorie foods, in combination with the previously found increased activation in response to high-calorie foods, which may reflect a specific preference for high-calorie foods, and may hamper SGLT2i-induced weight loss. Exenatide treatment increased activation in response to low-calorie foods. Combination treatment may lead to more favorable brain responses to low-calorie food cues, as we observed that the dapagliflozin-induced decreased response to low-calorie food pictures had disappeared.

## 1 Introduction

Sodium-glucose cotransporter-2 inhibitors (SGLT2i) and glucagon-like peptide-1 receptor agonists (GLP-1RA) are recently introduced drug classes for the treatment of type 2 diabetes (T2D). Besides their effects on glucose regulation, both reduce body weight.

Weight loss induced by SGLT2i is attributable to urinary glucose excretion. However, observed weight loss is less than expected based on the amount of calories excreted. Since energy expenditure remains unchanged with SGLT2i, the discrepancy between the observed and expected weight loss implies an increase in calorie intake (1, 2). Previously, we showed that dapagliflozin affects central satiety and reward centers, as we found that dapagliflozin increased regional brain responses to highly palatable food cues (3). Together with an observed increased appetite and carbohydrate intake, this could contribute to less weight loss than expected based on urinary calorie excretion. Other studies also suggested a specific increase in (craving for) high calorie/sweet foods with SGLT2i treatment (4–6).

The weight loss induced by GLP-1RAs is mainly attributable to suppressed appetite signaling in the brain and increased satiety, which leads to a reduced food intake *via* direct and indirect actions in the CNS. Interestingly, GLP-1 receptor activation selectively reduced intake of highly palatable, energy-dense food without affecting intake of standard diets in animals (7–10). In humans, GLP-1RA treatment is also associated with lower preferences for fatty, energy-dense foods (11–14). In addition, GLP-1RA treatment was associated with decreased activation in the insula, putamen, and amygdala in response to palatable food cues (15, 16), which may reflect a lower preference for energy-dense foods. However, it is unknown if GLP-1RA treatment is associated with an increased CNS activation to low-calorie food pictures, which may reflect a preference for healthy low-calorie foods.

Given their unique mechanisms of lowering glucose and body weight, the combination of SGLT2i and GLP-1RA appears promising (17–19). Recently we showed that in combination therapy, exenatide blunted the increased CNS activation observed with dapagliflozin in response to palatable food cues (3). However, the response to low-calorie foods has not been investigated.

Most studies investigated brain responses to high-calorie foods, as high-calorie foods induce greater hedonic responses in the brain. Interestingly, some studies also investigated brain responses to low-calorie food pictures and suggested that high- and low-calorie foods may be (partially) differently processed by the brain (20–22). In order to optimize treatments for obesity, we need a better understanding of how the intake of different nutrients is controlled, and how food choices are made. Therefore, the aim of the current study was to explore the effects of dapagliflozin, exenatide, and their combination on CNS activation in response to low-calorie food pictures.

## 2 Methods

### 2.1 Study Design

This was an exploratory analysis of the Dapagliflozin plus Exenatide on Central REgulation of Appetite in diabeteS typE 2 (DECREASE) study (3, 23) (NCT03361098). The study was approved by the Medical Ethics Committee of the Amsterdam University Medical Center, location Vumc, the Netherlands, and conducted in accordance with the Declaration of Helsinki and Good Clinical Practice. All patients provided informed consent.

### 2.2 Participants

Participants were recruited from our outpatient clinic database and by advertisement in local newspapers. We included men and postmenopausal women with type 2 diabetes, aged 18 to 75 years, with a stable body weight (<5% reported change during the previous 3 months), a BMI >25 kg/m^2^ who used metformin with or without sulfonylurea (stable dose for ≥ 3 months). HbA1c levels for participants treated with metformin monotherapy were 7%-10% and for metformin plus sulfonylurea 7.5%-10%. Exclusion criteria were a history of severe cardiovascular, renal, or liver disease, malignancies (excluding basal cell carcinoma), uncontrolled thyroid disease, the use of any centrally acting agent or oral glucocorticoids, substance abuse, neurological or psychiatric disease including eating disorders and depression, and MRI contra-indications. Written informed consent was obtained from all participants.

### 2.3 General Experimental Protocol

The design of this randomized double-blind placebo-controlled trial has been described in detail previously (3, 23). In summary, patients were randomized 1:1:1:1, performed by an independent trial pharmacist using computer-generated numbers, to 1. dapagliflozin 10 mg plus exenatide-matched placebo; 2. exenatide twice-daily 10 µg plus dapagliflozin-matched placebo; 3. dapagliflozin plus exenatide; or 4. placebo dapagliflozin plus placebo exenatide for 16 weeks. Exenatide (or placebo) was injected twice daily 15 to 30 minutes before breakfast and dinner, and was initiated at a dose of 5 µg, followed by a dose increase to 10 µg after 4 weeks, which was maintained until the end of the study. Dapagliflozin (or placebo) was taken once daily at 8 PM during the 16-week treatment period. To maintain blinding throughout the study, participants were treated in a double-dummy design. There was no difference in appearance between exenatide and placebo injections or dapagliflozin or placebo tablets.

There were three endpoint visits, at baseline, after 10 days, and 16 weeks of treatment. On these three visits, fMRI measurements and measurements of anthropometrics, blood pressure, and body composition were performed, and blood was drawn. The 10-day measurement was chosen to assess the effects of treatments on the CNS responses, independent of changes in body weight. The 16-week follow up time point was chosen because the largest reductions in HbA1c and body weight occur in the first ~16 weeks of treatment, and remain more or less stable thereafter (2, 24).

### 2.4 fMRI Protocol

The fMRI measurements were performed as described previously (3, 15, 16, 25). Briefly, pictures were presented in three runs comprising six blocks each: two blocks consisting of high-calorie food (sweet and savoury), two blocks of low-calorie food (fruits and vegetables), and two blocks of non-food items (e.g., trees, flowers, rocks, and bricks). Within each block seven pictures were presented for 2.5 s each, separated by a 0.5 s blank screen. Each block was followed by 9 s of grey blank screen with a fixation cross. Across each block and session, pictures were matched for shape and color

Imaging data were acquired using a 3.0 Tesla GE Signa HDxt scanner (General Electric, Milwaukee, WI, USA). Structural MRI was obtained using a T1-weighted sequence. fMRI data were acquired using an echo planar imaging T2-weighted blood oxygenation level–dependent sequence with 40 ascending slices per volume (3 mm thickness, 0 mm gap), which resulted in whole-brain coverage. Functional images were pre-processed with fMRIprep v1.2.3 (26), as described previously (3). T1-coregistered volumes were normalized to Montreal Neurological Institute space. Pre-processed data were analysed in the context of the general linear model with SPM12 (Wellcome Trust Centre for Neuroimaging, London, U.K.). At the first (single-subject) level, high-calorie food, low-calorie food, and non-food blocks were modeled. To assess if the effect of treatment on CNS activation related to viewing food pictures, and was food-cue specific, we computed the following contrasts at each time point: low-calorie vs non-food and high-calorie vs. non-food. To test our hypotheses, dapagliflozin, exenatide, and dapagliflozin plus exenatide were compared with placebo after 10 days and 16 weeks of treatment. In additional analyses, dapagliflozin was compared with dapagliflozin-exenatide.

*A priori* ROIs were determined based on previous studies (i.e., insula [including adjacent opercula], striatum [i.e., putamen and caudate nucleus], amygdala, and orbitofrontal cortex [OFC]), as these regions are consistently shown to be involved in responses to food cues and are part of the central reward circuits (27)). CNS activations were reported as significant when these survived family-wise error (FWE) correction for multiple comparisons (*P_FWE_* < 0.05) at the voxel level using small volume correction within the predefined ROIs, using 10-mm radius spheres (5-mm sphere for the amygdala) as described previously (15, 28).

## 3 Results

### 3.1 Baseline Characteristics

Of 68 patients, 65 patients completed the study protocol between September 2017 and March 2020 (3). One patient in the combination group discontinued treatment due to nausea, and one patient in the placebo group discontinued treatment due to personal reasons. One patient in the exenatide group experienced claustrophobia during baseline MRI measurements; this patient continued treatment without follow-up MRI measurements. Baseline characteristics were well balanced between treatment groups (**Table 1**) (3).

**Table 1:** Baseline characteristics.

	Dapagliflozin (n = 16)	Exenatide (n = 17)	Dapagliflozin + Exenatide (n = 16)	Placebo (n = 16)
Age (years)	64 (8·4)	65 (5·8)	64 (7·4)	60·9 (7·2)
Female [n (%)]	4 (25)	6 (35·3)	4 (25)	4 (25·0)
Weight (kg)	97·8 (15·4)	96·6 (13·3)	93·6 (13·4)	99·1 (21·9)
BMI (kg/m^2^)	31·7 (3·3)	32·7 (5·1)	30·9 (3·4)	31·5 (5·9)
Body fat (%)	34·9 (5·5)	38·6 (8·6)	34·9 (6·2)	34·9 (7·4)
Diabetes duration (years)	8·0 [5·5,13·5]	10·0 [6,18]	7·0 [5,12·8]	9·5 [7,10·5]
Fasting glucose (mmol/l)	8·7 (1·5)	9·9 (1·9)	10·7 (3·6)	9·5 (3·0)
HbA1c (% )(mmol/mol)	7·8 (0·6) 61·3 (6·1)	7·9 (0·8) 65·0 (11·1)	8·0 (1·3) 63·5 (14·5)	8·0 (0·95) 64·7 (11·7)
eGFR (ml/min/1·73m^2^)	83·4 (14·6)	83·2 (13·7)	88·8 (10·6)	87·8 (11·2)
Use of [n (%)]				
*Metformin*	16 (100)	17 (100)	16 (100)	16 (100)
*SU derivative*	5 (31·3)	6 (35·3)	3 (18·8)	8 (50·0)
*Beta blocker*	4 (25·0)	4 (23·5)	3 (18·8)	2 (12·5)
*Statin*	12 (75·0)	14 (82·4)	12 (75·0)	14 (87·5)
*Anti-coagulant*	4 (25·0)	4 (23·5)	5 (31·3)	1 (6·3)
*RAS inhibition*	5 (31·3)	12 (70·6)	10 (66·7)	9 (56·3)
*ACE inhibitor*	2 (40·0)	8 (47·1)	7 (70·0)	6 (66·7)
*ARB*	3 (60·0)	4 (23·5)	3 (30·0)	3 (30·0)

Data are means ± SD or median [interquartile range] for continuous metrics, and number (percent) for categorical characteristics. BMI, body mass index; eGFR, estimated glomerular filtration rate; SU, Sulfonylurea; ACE, angiotensin converting enzyme; ARB, angiotensin-II receptor blocker.

### 3.2 Anthropometrics and Glycemic Control

As previously published, compared with placebo, dapagliflozin reduced body weight by -2.5 ± 0.5 kg (p<0.001), exenatide by -1.4 ± 0.5 kg (p<0.01), and the combination by -2.8 ± 0.5 kg (p<0.001), after 16 weeks of treatment (3). In addition, compared with placebo, dapagliflozin reduced HbA1c by 0.5 ± 0.19% (5 mmol/mol, p<0.01), exenatide by 0.8 ± 0.18% (8.4 mmol/mol, p<0.001), and the combination by -1.2 ± 0.19% (11.9 mmol/mol, p<0.001) after 16 weeks (3).

### 3.3 CNS Responses to Food Pictures

#### 3.3.1 Dapagliflozin Compared With Placebo

**Figure 1:**
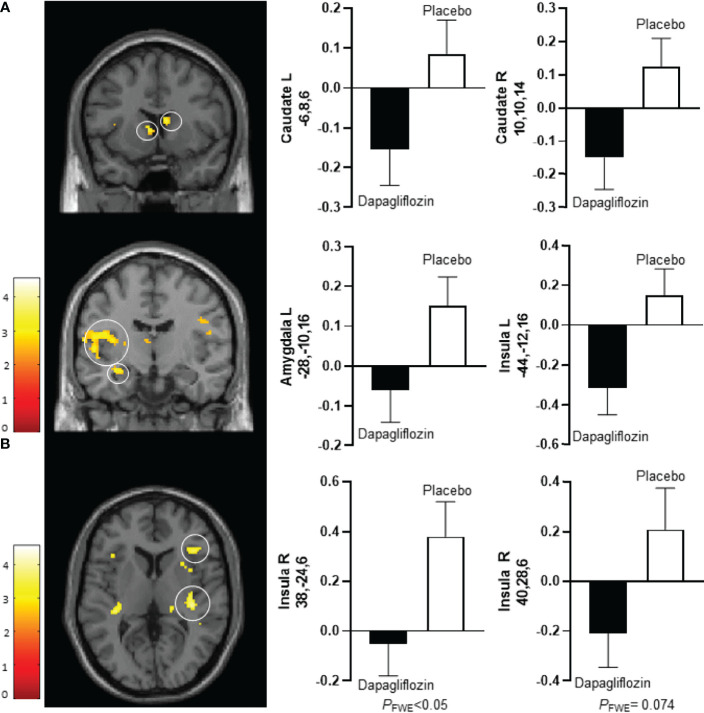
Average differences in CNS activation between dapagliflozin (black bar) and placebo (white bar). **(A)** Axial slice showing average differences between dapagliflozin (black bar) and placebo (white bar) after 10 days of treatment in the left caudate nucleus (*P*_FWE_ < 0.05, T = 3.7) (upper left panel), the right caudate nucleus (*P*_FWE_ < 0.05, T = 3.5) (upper right panel), the left amygdala (*P*_FWE_ < 0.05, T = 3.1) (middle left panel), and the left insula (*P*_FWE_ < 0.05, T = 3.6) (middle right panel) in response to the viewing of low-calorie versus non-food pictures. **(B)**. Horizontal slice showing average differences between dapagliflozin (black bar) and placebo (white bar) after 16 weeks of treatment in the right insula (left panel: *P*_FWE_ < 0.05, T = 3.5), and non-statistically significant in the right insula (right panel: *P*_FWE_ = 0.074, T = 3.2) in response to the viewing of low-calorie food pictures versus non-food pictures. The left side of the brain slices is the left side of the brain. The color scale reflects the T value of the functional activity. Results are presented at the threshold of *P* < 0.05, familywise error (FWE) corrected on cluster extent within the regions of interest using small volume correction (10 mm sphere; 5 mm sphere for the amygdala). In the graphs, blood oxygen level-dependent (BOLD) signal intensity (effect size) is plotted (arbitrary unites), mean and SEM. The numbers on the y-axes of the bar graphs are the x,y,z, coordinates of the peak voxel of the observed difference in Montreal Neurological Institute (MNI) space. R, right; L, left.

After 10 days dapagliflozin compared with placebo decreased activity in the bilateral caudate nucleus (right, T 3.5, *P*‐_FWE_ 0.035; left, T 3.7, *P*‐_FWE_ 0.023), left insula (T 3.6, *P*‐_FWE_ 0.027), and left amygdala (T 3.1, *P*‐_FWE_ 0.021) in response to low-calorie pictures (**Table 2**; **Figure 1A**). After 16 weeks of treatment, dapagliflozin compared with placebo reduced activity in the right insula (T 3.5, *P*‐_FWE_ 0.041) in response to low-calorie pictures (**Table 2**; **Figure 1B**).

**Table 2:** Effects of dapagliflozin, exenatide and the combination of dapagliflozin and exenatide on brain responses to low-calorie food pictures.

-Group/comparison	Time point	Contrast	Region	Side	Cluster	T	*P*‐_FWE_	MNI coordinates (x, y, z)
Placebo > Dapagliflozin								
	10 days	Low-calorie > non-food	Caudate nucleus Caudate nucleus Insula Amygdala	R L L L	40 28 183 32	3.5 3.7 3.6 3.1	0.035 0.023 0.027 0.021	10,10,14 -6,8,6 -44,-12,16 -28,-10,-16
	16 weeks	Low-calorie > non-food	Insula *Insula*	R *R*	75 *39*	3.5 *3.2*	0.041 *0.076*	38,-24,6 *40,28,6*
Exenatide > Placebo								
	10 days	Low-calorie > non-food	Putamen *Insula*	R *R*	31 *122*	3.6 *3.3*	0.027 *0.057*	32,0,6 *42,6,-8*
	16 weeks	Low-calorie > non-food	NS	NS	NS	NS	NS	NS
Combination > Placebo								
	10 days	Low-calorie > non-food	NS	NS	NS	NS	NS	NS
	16 weeks	Low-calorie > non-food	NS	NS	NS	NS	NS	NS
Combination > Dapagliflozin								
	10 days	Low-calorie > non-food	Insula Insula Insula Insula Amygdala	R R L L R	141 150 144 213 17	4.3 3.8 4.3 4.2 2.8	0.006 0.016 0.004 0.004 0.043	46,-4,16 32,-22,12 -42,-14,16 -36,-32,18 28,-8,-14
	16 weeks	Low-calorie > non-food	*Putamen*	*L*		*3.1*	*0.065*	*-32,2,0*

This table describes the areas where significant differences in activations were observed with dapagliflozin, exenatide, and combination of dapagliflozin plus exenatide compared with placebo treatment. For each comparison, the contrast (activation during low-calorie > non-food pictures) is presented. The areas with significant differences are listed, including the cluster size of this effect, the T value, and the FWE corrected p-value after small volume correction. The last column describes the coordinates of the peak voxel of the observed difference in MNI space. For completeness non-significant results in regions of interest are showed in grey. Combination, dapagliflozin plus exenatide; T, T-value; P‐_FWE_, P‐value family‐wise error corrected for multiple comparisons on the basis of cluster extent (small volume correction); R, right; L, left; MNI, Montreal Neurological Institute coordinates in mm, which represents the exact three dimensional location [x=horizontal, y=horizontal, z= vertical axis in mm distance from the origin (which is the intersection of the three axis)] in the brain of the significant activation; NS, indicating that there were no statistical significant results for this comparison.

#### 3.3.2 Exenatide Compared With Placebo

**Figure 2:**
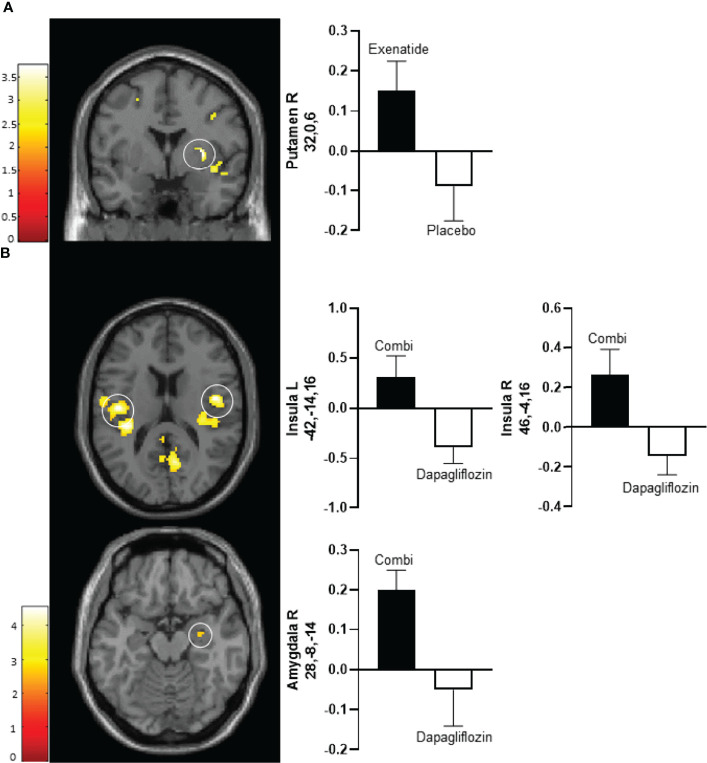
Average differences in CNS activation after 10 days of treatment. **(A)** Axial slice showing average differences between exenatide (black bar) and placebo (white bar) in the right putamen (*P*_FWE_ < 0.01, T=3.2) (upper panel) in response to the viewing of low-calorie versus non-food pictures. **(B)** Horizontal slice showing average differences between dapagliflozin plus exenatide (combi) (black bar) and dapagliflozin (white bar) in the left insula (*P*_FWE_ < 0.01, T = 4.3) (middle left panel), right insula (*P*_FWE_ < 0.01, T = 4.3) (middle right panel), and right amygdala (*P*_FWE_ < 0.05, T = 2.8) (bottom panel) in response to the viewing of low-calorie food pictures versus non-food pictures. The left side of the brain slices is the left side of the brain. The color scale reflects the T value of the functional activity. Results are presented at the threshold of *P* < 0.05, familywise error (FWE) corrected on cluster extent within the regions of interest using small volume correction (10 mm sphere; 5 mm sphere for the amygdala). In the graphs, blood oxygen level-dependent (BOLD) signal intensity (effect size) is plotted (arbitrary unites), mean and SEM. The numbers on the y-axes of the bar graphs are the x,y,z, coordinates of the peak voxel of the observed difference in Montreal Neurological Institute (MNI) space. Combi, dapagliflozin plus exenatide; R, right; L, left.

After 10 days exenatide compared with placebo increased activity in the right putamen (T 3.6, *P*‐_FWE_ 0.027) and tended to increase activity in right insula (T 3.3, *P*‐_FWE_ 0.057) in response to low-calorie pictures (**Table 2**; **Figure 2A**). After 16 weeks exenatide compared with placebo had no effect on brain activation in response to low-calorie pictures.

#### 3.3.3 Dapagliflozin-Exenatide Compared With Placebo

Dapagliflozin-exenatide compared with placebo had no effect on brain activation in response to low-calorie pictures.

#### 3.3.4 Dapagliflozin-Exenatide Compared With Dapagliflozin

After 10 days of treatment, dapagliflozin-exenatide compared with dapagliflozin increased activity to low-calorie food pictures in bilateral insula (right T 4.3, *P*‐_FWE_ = 0.006, T 3.8, *P*‐_FWE_ = 0.016; left T=4.3, *P*‐_FWE_ = 0.004, T=4.2, *P*‐_FWE_ = 0.006) and right amygdala (T 2.8, *P*‐_FWE_ = 0.043) (**Table 2**; **Figure 2B**). After 16 weeks combination therapy tended to increase activity in the left putamen (T 3.1, *P*‐_FWE_ 0.065) in response to low-calorie pictures (**Table 2**).

## 4 Discussion

This is the first study investigating CNS activation in response to low-calorie food pictures with dapagliflozin, exenatide, or combination treatment. Previously we demonstrated that dapagliflozin increased appetite and carbohydrate intake, together with increased CNS activation in response to high-calorie (hedonic) food pictures after short-term treatment (3). Here, we demonstrated that the neural responses to food pictures with dapagliflozin may be food cue specific, as dapagliflozin was associated with reduced activation in response to low-calorie (non-hedonic) food pictures after short (10 days) and longer-term (16 weeks) treatment. In addition, exenatide was associated with increased CNS responses to low-calorie food pictures, while the combination of dapagliflozin and exenatide showed no effect on low-calorie food pictures.

Previously, we showed that the presumed SGLT2i-induced hyperphagia (1, 2, 29) may be specific for high-calorie/sweet foods, as we and others found an increase in carbohydrate intake (3, 30), or sugar intake (5). In line with this, we previously observed that dapagliflozin increased CNS activation in response to high-calorie foods, which may reflect the neurophysiological correlate for the observed preference for high-calorie/sweet foods. Interestingly, this is the first study that also demonstrated decreased CNS activation in response to low-calorie food pictures, which may reflect a specific decreased preference for low-calorie foods. This may have clinical consequences as a specific dislike for low-calorie foods in combination with a preference for high-calorie foods and may hamper SGLT2i-induced weight loss.

Some studies suggested a specific dislike for calorie-dense/sweet foods with GLP-1RA treatment (11–14). Here, we are the first to demonstrate that exenatide increased CNS activation in response to low-calorie food pictures, while we previously demonstrated that liraglutide (16) and exenatide (3) reduced CNS activation to high-calorie food pictures. Together, these findings suggest an increased preferential response to low-calorie foods. Interestingly, in line with these results, a recent observational study found a positive correlation between endogenous circulating GLP-1 and dorsal striatal responsiveness to low-calorie food cues, and a negative correlation between GLP-1 and dorsal striatal responding to high-calorie food cues (31).

After combining dapagliflozin with exenatide, the dapagliflozin-induced decrease in CNS activation in response to low-calorie food pictures had disappeared, presumably due to activation increasing effects of exenatide. These findings complement the findings of CNS responsiveness to high-calorie food pictures, as we found no effect of dapagliflozin plus exenatide, while dapagliflozin increased responses and exenatide decreased CNS responses to high-calorie food pictures (3). Together, this suggests that exenatide can (partially) alter the food preferences induced by dapagliflozin, which may contribute to more weight loss with the combination.

*“*Effects of treatments compared with placebo were observed in putamen, caudate nucleus, insula and amygdala. These brain regions are part of a complex reward circuitry (32). The insula receives gustatory and visceral afferents, is involved in taste memory and is also involved in the rewarding aspects of food (33). The insula modulates the activity of the other brain regions, based on homeostatic signals, and translates these internal signals into subjective feelings such as the urge to eat (34). The putamen is engaged in the reward processing and conditioning, and activation in the putamen is associated with predicting future weight gain (35). The amygdala is involved in the association of cues with reward and emotional learning (36).

“SGLTs are glucose transporters in the intestine, kidney, and also in the brain (37–39). The expression of SGLT2 in brain is low, and therefore its physiological function remains questionable (40). Dapagliflozin has a higher affinity for SGLT2 (41), but could potentially also affect SGLT1. SGLT1 is expressed in neurons throughout the brain, showing high expression in regions that are involved in learning, regulation of feeding behavior, energy expenditure and glucose homeostasis (40). A study in rats demonstrated a direct effect of SGLT2i on the CNS as central administration of SGLT2i increased food intake (42). Metabolic adaptions may also play a role to compensate for the chronic calorie loss. “Further research to investigate the exact mechanism of how SGLT2 inhibition affects the CNS is needed.”

Although the design of the trial is one of its major strengths, the study was not specifically designed for this exploratory analysis and should therefore be considered as hypothesis generating. We assessed appetite scores, but we did not administer questionnaires determining preferences for specific (aspects of) food (i.e., sweet, fatty, savory, salty). Food intake was measured with an ad libitum lunch buffet, but daily food intake was not measured. However, measuring food intake is a major challenge in free-living individuals, and people with obesity are known to underreport their intake (43). Although we investigated changes in CNS activation in predefined ROIs which are consistently shown to be involved in responses to food cues, and are part of the central reward circuits, it would have been of interest to include regions for homeostatic control of feeding such as the nucleus tractus solitarii in the brain stem and the hypothalamus. However, visualization of both using fMRI is challenging due to their size and location. Further research primarily investigating the neurophysiological correlates for food preferences, with sufficient participants, and stringent measurements of food intake (i.e., weighed food intake) should be performed to confirm our results.

In conclusion, previously we showed that dapagliflozin treatment increased activation in feeding regulation areas in responses to high-calorie foods, which suggests that the hyperphagia with dapagliflozin treatment may be specific for high-calorie/sweet foods. Here, we also demonstrated a decreased activation in response to low-calorie foods in people with obesity and T2D, which may reflect a specific decreased preference for low-calorie foods. This may have clinical consequences as a specific dislike for low-calorie foods in combination with a preference for high-calorie foods may hamper the weight loss of SGLT2i. In contrast, exenatide treatment resulted in an increased activation in response to low-calorie foods. The combination of dapagliflozin plus exenatide may lead to more favorable brain responses to food cues, as we observed that the dapagliflozin-induced decrease to low-calorie food pictures had disappeared. These findings provide further insight in the appetite and weight lowering effects with SGLT2i and GLP-1RAs alone and in combination and may contribute to optimizing weight loss.

## Data Availability Statement

The raw data supporting the conclusions of this article will be made available by the authors, without undue reservation.

## Ethics Statement

The studies involving human participants were reviewed and approved by Medisch etische toetsings commissie VUmc (Metc VUmc). The patients/participants provided their written informed consent to participate in this study.

## Author Contributions

CR designed the study, conducted the experiments, performed the data analysis, and wrote the article. DV designed the fMRI paradigm, performed the data analysis, and contributed to writing the article. MN contributed to writing the article. RI designed the study and the fMRI paradigm, performed the data analysis, and wrote the manuscript. All authors have seen and approved the final version of the manuscript. CR and RI are the guarantors of this work and, as such, had full access to all the data in the study and take responsibility for the integrity of the data and the accuracy of the data analysis. All authors contributed to the article and approved the submitted version.

## Funding

This work was funded by an investigator initiated grant from AstraZeneca (ESR-16-11865). The funder had no role in the study design, data analyses or interpretation, or drafting of the manuscript, nor in the decision to submit the manuscript for publication.

## Conflict of Interest

RI is principal investigator of studies sponsored by research grants from AstraZeneca, Eli Lilly & Co., and Novo Nordisk. MN is supported by a personal ZONMW VICI grant 2020 (09150182010020) and received an unrestricted grant from AstraZeneca and serves on the Scientific Advisory Board of Caelus Pharmaceuticals, the Netherlands, and Kaleido, USA. All authors declare they have not received any fees personally in connection with the roles described above, as all honoraria were paid to their employer (Amsterdam University Medical Centers, location VUmc). None of these potential conflicts of interest are relevant to this article.

The remaining authors declare that the research was conducted in the absence of any commercial or financial relationships that could be construed as a potential conflict of interest

## Publisher’s Note

All claims expressed in this article are solely those of the authors and do not necessarily represent those of their affiliated organizations, or those of the publisher, the editors and the reviewers. Any product that may be evaluated in this article, or claim that may be made by its manufacturer, is not guaranteed or endorsed by the publisher.

## References

[1] Polidori D Sanghvi A Seeley RJ Hall KD. How Strongly Does Appetite Counter Weight Loss? Quantification of the Feedback Control of Human Energy Intake. Obes (Silver Spring) (2016) 24(11):2289–95. doi: 10.1002/oby.21653 PMC5108589 27804272

[2] Ferrannini G Hach T Crowe S Sanghvi A Hall KD Ferrannini E. Energy Balance After Sodium-Glucose Cotransporter 2 Inhibition. Diabetes Care (2015) 38(9):1730–5. doi: 10.2337/dc15-0355 PMC4542276 26180105

[3] van Ruiten CC Veltman DJ Schrantee A van Bloemendaal L Barkhof F Kramer MHH. Effects of Dapagliflozin and Combination Therapy With Exenatide on Food-Cue Induced Brain Activation in Patients With Type 2 Diabetes. J Clin Endocrinol Metab (2022) 1–10. doi: 10.1210/clinem/dgac043 35134184 PMC9113812

[4] Bertran E Berlie HD Nixon A Jaber L. Does Dapagliflozin Affect Energy Intake and Appetite? A Randomized, Controlled Exploratory Study in Healthy Subjects. Clin Pharmacol Drug Dev (2019) 8(1):119–25. doi: 10.1002/cpdd.461 29723443

[5] Horie I Abiru N Hongo R Nakamura T Ito A Haraguchi A. Increased Sugar Intake as a Form of Compensatory Hyperphagia in Patients With Type 2 Diabetes Under Dapagliflozin Treatment. Diabetes Res Clin Pract (2018) 135:178–84. doi: 10.1016/j.diabres.2017.11.016 29162514

[6] Matsuba I Kanamori A Takihata M Takai M Maeda H Kubota A. Canagliflozin Increases Calorie Intake in Type 2 Diabetes Without Changing the Energy Ratio of the Three Macronutrients: CANA-K Study. Diabetes Technol Ther (2020) 22(3):228–34. doi: 10.1089/dia.2019.0372 32013567

[7] Alhadeff AL Rupprecht LE Hayes MR. GLP-1 Neurons in the Nucleus of the Solitary Tract Project Directly to the Ventral Tegmental Area and Nucleus Accumbens to Control for Food Intake. Endocrinology (2012) 153(2):647–58. doi: 10.1210/en.2011-1443 PMC3275387 22128031

[8] Mietlicki-Baase EG Ortinski PI Reiner DJ Sinon CG McCutcheon JE Pierce RC. Glucagon-Like Peptide-1 Receptor Activation in the Nucleus Accumbens Core Suppresses Feeding by Increasing Glutamatergic AMPA/kainate Signaling. J Neurosci (2014) 34(20):6985–92. doi: 10.1523/JNEUROSCI.0115-14.2014 PMC4019807 24828651

[9] Mietlicki-Baase EG Ortinski PI Rupprecht LE Olivos DR Alhadeff AL Pierce RC. The Food Intake-Suppressive Effects of Glucagon-Like Peptide-1 Receptor Signaling in the Ventral Tegmental Area Are Mediated by AMPA/kainate Receptors. Am J Physiol Endocrinol Metab (2013) 305(11):E1367-74. doi: 10.1152/ajpendo.00413.2013 24105414 PMC3882373

[10] Wang XF Liu JJ Xia J Liu J Mirabella V Pang ZP. Endogenous Glucagon-Like Peptide-1 Suppresses High-Fat Food Intake by Reducing Synaptic Drive Onto Mesolimbic Dopamine Neurons. Cell Rep (2015) 12(5):726–33. doi: 10.1016/j.celrep.2015.06.062 PMC4860285 26212334

[11] Blundell J Finlayson G Axelsen M Flint A Gibbons C Kvist T. Effects of Once-Weekly Semaglutide on Appetite, Energy Intake, Control of Eating, Food Preference and Body Weight in Subjects With Obesity. Diabetes Obes Metab (2017) 19(9):1242–51. doi: 10.1111/dom.12932 PMC5573908 28266779

[12] Brindisi MC Brondel L Meillon S Barthet S Grall S Fenech C. Proof of Concept: Effect of GLP-1 Agonist on Food Hedonic Responses and Taste Sensitivity in Poor Controlled Type 2 Diabetic Patients. Diabetes Metab Syndr (2019) 13(4):2489–94. doi: 10.1016/j.dsx.2019.06.021 31405666

[13] Kadouh H Chedid V Halawi H Burton DD Clark MM Khemani D. GLP-1 Analog Modulates Appetite, Taste Preference, Gut Hormones, and Regional Body Fat Stores in Adults With Obesity. J Clin Endocrinol Metab (2020) 105(5):1552–63. doi: 10.1210/clinem/dgz140 PMC7105351 31665455

[14] Friedrichsen M Breitschaft A Tadayon S Wizert A Skovgaard D. The Effect of Semaglutide 2.4 Mg Once Weekly on Energy Intake, Appetite, Control of Eating, and Gastric Emptying in Adults With Obesity. Diabetes Obes Metab (2021) 23(3):754–62. doi: 10.1111/dom.14280 PMC7898914 33269530

[15] van Bloemendaal L IJ RG Ten Kulve JS Barkhof F Konrad RJ Drent ML. GLP-1 Receptor Activation Modulates Appetite- and Reward-Related Brain Areas in Humans. Diabetes (2014) 63(12):4186–96. doi: 10.2337/db14-0849 25071023

[16] Ten Kulve JS Veltman DJ van Bloemendaal L Barkhof F Drent ML Diamant M. Liraglutide Reduces CNS Activation in Response to Visual Food Cues Only After Short-Term Treatment in Patients With Type 2 Diabetes. Diabetes Care (2016) 39(2):214–21. doi: 10.2337/dc15-0772 26283736

[17] Frias JP Guja C Hardy E Ahmed A Dong F Ohman P. Exenatide Once Weekly Plus Dapagliflozin Once Daily Versus Exenatide or Dapagliflozin Alone in Patients With Type 2 Diabetes Inadequately Controlled With Metformin Monotherapy (DURATION-8): A 28 Week, Multicentre, Double-Blind, Phase 3, Randomised Controlled Trial. Lancet Diabetes Endocrinol (2016) 4(12):1004–16. doi: 10.1016/S2213-8587(16)30267-4 27651331

[18] Zinman B Bhosekar V Busch R Holst I Ludvik B Thielke D. Semaglutide Once Weekly as Add-on to SGLT-2 Inhibitor Therapy in Type 2 Diabetes (SUSTAIN 9): A Randomised, Placebo-Controlled Trial. Lancet Diabetes Endocrinol (2019) 7(5):356–67. doi: 10.1016/S2213-8587(19)30066-X 30833170

[19] Ludvik B Frias JP Tinahones FJ Wainstein J Jiang H Robertson KE. Dulaglutide as Add-on Therapy to SGLT2 Inhibitors in Patients With Inadequately Controlled Type 2 Diabetes (AWARD-10): A 24-Week, Randomised, Double-Blind, Placebo-Controlled Trial. Lancet Diabetes Endocrinol (2018) 6(5):370–81. doi: 10.1016/S2213-8587(18)30023-8 29483060

[20] Killgore WD Yurgelun-Todd DA. Developmental Changes in the Functional Brain Responses of Adolescents to Images of High and Low-Calorie Foods. Dev Psychobiol (2005) 47(4):377–97. doi: 10.1002/dev.20099 16284969

[21] Rothemund Y Preuschhof C Bohner G Bauknecht HC Klingebiel R Flor H. Differential Activation of the Dorsal Striatum by High-Calorie Visual Food Stimuli in Obese Individuals. Neuroimage (2007) 37(2):410–21. doi: 10.1016/j.neuroimage.2007.05.008 17566768

[22] Fletcher PC Napolitano A Skeggs A Miller SR Delafont B Cambridge VC. Distinct Modulatory Effects of Satiety and Sibutramine on Brain Responses to Food Images in Humans: A Double Dissociation Across Hypothalamus, Amygdala, and Ventral Striatum. J Neurosci (2010) 30(43):14346–55. doi: 10.1523/JNEUROSCI.3323-10.2010 PMC3447260 20980590

[23] van Ruiten CC van der Aart-van der Beek AB RG IJ Nieuwdorp M Hoogenberg K van Raalte DH. Effect of Exenatide Twice Daily and Dapagliflozin, Alone and in Combination, on Markers of Kidney Function in Obese Patients With Type 2 Diabetes: A Prespecified Secondary Analysis of a Randomized Controlled Clinical Trial. Diabetes Obes Metab (2021) 23:1851–8. doi: 10.1111/dom.14410 PMC8360098 33908691

[24] Jabbour SA Frias JP Ahmed A Hardy E Choi J Sjostrom CD. Efficacy and Safety Over 2 Years of Exenatide Plus Dapagliflozin in the DURATION-8 Study: A Multicenter, Double-Blind, Phase 3, Randomized Controlled Trial. Diabetes Care (2020) 43(10):2528–36. doi: 10.2337/dc19-1350. PMC7510043 32816874

[25] Doornweerd S De Geus EJ Barkhof F Van Bloemendaal L Boomsma DI Van Dongen J. Brain Reward Responses to Food Stimuli Among Female Monozygotic Twins Discordant for BMI. Brain Imaging Behav (2018) 12(3):718–27. doi: 10.1007/s11682-017-9711-1 PMC5990553 28597337

[26] Esteban O Markiewicz CJ Blair RW Moodie CA Isik AI Erramuzpe A. Fmriprep: A Robust Preprocessing Pipeline for Functional MRI. Nat Methods (2019) 16(1):111–6. doi: 10.1038/s41592-018-0235-4 PMC6319393 30532080

[27] Stoeckel LE Weller RE Cook EW 3rd Twieg DB Knowlton RC Cox JE. Widespread Reward-System Activation in Obese Women in Response to Pictures of High-Calorie Foods. Neuroimage (2008) 41(2):636–47. doi: 10.1016/j.neuroimage.2008.02.031 18413289

[28] ten Kulve JS Veltman DJ van Bloemendaal L Barkhof F Deacon CF Holst JJ. Endogenous GLP-1 Mediates Postprandial Reductions in Activation in Central Reward and Satiety Areas in Patients With Type 2 Diabetes. Diabetologia (2015) 58(12):2688–98. doi: 10.1007/s00125-015-3754-x PMC4630252 26385462

[29] Devenny JJ Godonis HE Harvey SJ Rooney S Cullen MJ Pelleymounter MA. Weight Loss Induced by Chronic Dapagliflozin Treatment is Attenuated by Compensatory Hyperphagia in Diet-Induced Obese (DIO) Rats. Obes (Silver Spring) (2012) 20(8):1645–52. doi: 10.1038/oby.2012.59 22402735

[30] Perkins BA Cherney DZ Partridge H Soleymanlou N Tschirhart H Zinman B. Sodium-Glucose Cotransporter 2 Inhibition and Glycemic Control in Type 1 Diabetes: Results of an 8-Week Open-Label Proof-of-Concept Trial. Diabetes Care (2014) 37(5):1480–3. doi: 10.2337/dc13-2338 24595630

[31] Jones S Luo S Dorton HM Yunker AG Angelo B Defendis A. Obesity and Dietary Added Sugar Interact to Affect Postprandial GLP-1 and Its Relationship to Striatal Responses to Food Cues and Feeding Behavior. Front Endocrinol (Lausanne) (2021) 12:638504. doi: 10.3389/fendo.2021.638504 33868172 PMC8044510

[32] Chen J Papies EK Barsalou LW. A Core Eating Network and Its Modulations Underlie Diverse Eating Phenomena. Brain Cogn (2016) 110:20–42. doi: 10.1016/j.bandc.2016.04.004 27156016

[33] Frank S Kullmann S Veit R. Food Related Processes in the Insular Cortex. Front Hum Neurosci (2013) 7:499. doi: 10.3389/fnhum.2013.00499 23986683 PMC3750209

[34] Craig AD. How Do You Feel–Now? The Anterior Insula and Human Awareness. Nat Rev Neurosci (2009) 10(1):59–70. doi: 10.1038/nrn2555 19096369

[35] Burger KS Stice E. Greater Striatopallidal Adaptive Coding During Cue-Reward Learning and Food Reward Habituation Predict Future Weight Gain. Neuroimage (2014) 99:122–8. doi: 10.1016/j.neuroimage.2014.05.066 PMC4142439 24893320

[36] Baxter MG Murray EA. The Amygdala and Reward. Nat Rev Neurosci (2002) 3(7):563–73. doi: 10.1038/nrn875 12094212

[37] Wright EM Loo DD Hirayama BA. Biology of Human Sodium Glucose Transporters. Physiol Rev (2011) 91(2):733–94. doi: 10.1152/physrev.00055.2009 21527736

[38] Yu AS Hirayama BA Timbol G Liu J Diez-Sampedro A Kepe V. Regional Distribution of SGLT Activity in Rat Brain *In Vivo* . Am J Physiol Cell Physiol (2013) 304(3):C240–7. doi: 10.1152/ajpcell.00317.2012 PMC3566441 23151803

[39] Chiba Y Sugiyama Y Nishi N Nonaka W Murakami R Ueno M. Sodium/glucose Cotransporter 2 Is Expressed in Choroid Plexus Epithelial Cells and Ependymal Cells in Human and Mouse Brains. Neuropathology (2020) 40(5):482–91. doi: 10.1111/neup.12665 PMC7587001 32488949

[40] Koepsell H. Glucose Transporters in Brain in Health and Disease. Pflugers Arch (2020) 472(9):1299–343. doi: 10.1007/s00424-020-02441-x PMC7462931 32789766

[41] van Bommel EJ Muskiet MH Tonneijck L Kramer MH Nieuwdorp M van Raalte DH. SGLT2 Inhibition in the Diabetic Kidney-From Mechanisms to Clinical Outcome. Clin J Am Soc Nephrol (2017) 12(4):700–10. doi: 10.2215/CJN.06080616 PMC5383382 28254770

[42] Takeda K Ono H Ishikawa K Ohno T Kumagai J Ochiai H. Central Administration of Sodium-Glucose Cotransporter-2 Inhibitors Increases Food Intake Involving Adenosine Monophosphate-Activated Protein Kinase Phosphorylation in the Lateral Hypothalamus in Healthy Rats. BMJ Open Diabetes Res Care (2021) 9(1)e002104. doi: 10.1136/bmjdrc-2020-002104 PMC8061802 33879516

[43] Wehling H Lusher J. People With a Body Mass Index 30 Under-Report Their Dietary Intake: A Systematic Review. J Health Psychol (2019) 24(14):2042–59. doi: 10.1177/1359105317714318 28810493

